# Dissection of Root Transcriptional Responses to Low pH, Aluminum Toxicity and Iron Excess Under Pi-Limiting Conditions in Arabidopsis Wild-Type and *stop1* Seedlings

**DOI:** 10.3389/fpls.2020.01200

**Published:** 2020-09-29

**Authors:** Jonathan Odilón Ojeda-Rivera, Araceli Oropeza-Aburto, Luis Herrera-Estrella

**Affiliations:** ^1^ Laboratorio Nacional de Genómica para la Biodiversidad (UGA) del Centro de Investigación y de Estudios Avanzados del IPN, Irapuato, México; ^2^ Plant and Soil Science Department, Institute of Genomics for Crop Abiotic Stress Tolerance, Texas Tech University, Lubbock, TX, United States

**Keywords:** root, transcriptome, acid soil, aluminum, iron, gene regulation, phosphate, combinatorial regulation

## Abstract

Acidic soils constrain plant growth and development in natural and agricultural ecosystems because of the combination of multiple stress factors including high levels of Fe^3+^, toxic levels of Al^3+^, low phosphate (Pi) availability and proton rhizotoxicity. The transcription factor SENSITIVE TO PROTON RHIZOTOXICITY (STOP1) has been reported to underlie root adaptation to low pH, Al^3+^ toxicity and low Pi availability by activating the expression of genes involved in organic acid exudation, regulation of pH homeostasis, Al^3+^ detoxification and root architecture remodeling in *Arabidopsis thaliana*. However, the mechanisms by which STOP1 integrates these environmental signals to trigger adaptive responses to variable conditions in acidic soils remain to be unraveled. It is unknown whether STOP1 activates the expression of a single set of genes that enables root adaptation to acidic soils or multiple gene sets depending on the combination of different types of stress present in acidic soils. Previous transcriptomic studies of *stop1* mutants and wild-type plants analyzed the effect of individual types of stress prevalent in acidic soils. An integrative study of the transcriptional regulation pathways that are activated by STOP1 under the combination of major stresses common in acidic soils is lacking. Using RNA-seq, we performed a transcriptional dissection of wild-type and *stop1* root responses, individually or in combination, to toxic levels of Al^3+^, low Pi availability, low pH and Fe excess. We show that the level of STOP1 is post-transcriptionally and coordinately upregulated in the roots of seedlings exposed to single or combined stress factors. The accumulation of STOP1 correlates with the transcriptional activation of stress-specific and common gene sets that are activated in the roots of wild-type seedlings but not in *stop1*. Our data indicate that perception of low Pi availability, low pH, Fe excess and Al toxicity converges at two levels *via* STOP1 signaling: post-translationally through the regulation of STOP1 turnover, and transcriptionally, *via* the activation of STOP1-dependent gene expression that enables the root to better adapt to abiotic stress factors present in acidic soils.

## Introduction

Acidic soils prevalent in tropical and subtropical areas of the planet represent up to 40% of the world’s arable land and constrain plant development and productivity in both natural and agricultural ecosystems (von Uexküll and Mutert, 1995). At a pH value of 5.5 or below, acidic pH compromises plant development because of a combination of two major stresses: lower nutrient availability, predominantly low phosphate (Pi) availability, and an increased availability of toxic cations, H^+^, Al^3+^ and Fe^3+^, which are detrimental for root development (Kochian et al., 2004; Kobayashi et al., 2013; Das et al., 2017). Given the agronomic relevance of acidic soils, research groups around the globe have focused on the characterization of the genetic, biochemical, physiological and morphological responses that allow plants to better adapt to acidic soils (see Magalhaes et al., 2018 for review).

Several studies in the model plant *Arabidopsis* *thaliana* have highlighted the role of the Cys_2_-His_2_-type zinc finger transcription factor SENSITIVE TO PROTON RHIZOTOXICITY 1 (STOP1) in protecting the root from the conditions present in acidic soils. When *stop1* mutants were first isolated, it was discovered that these mutant seedlings were hypersensitive to both H^+^ and Al^3+^ rhizotoxicities (Iuchi et al., 2007). STOP1 confers root tolerance to Al toxicity by promoting malate exudation by upregulating the expression of the malate efflux transporter ALUMINUM ACTIVATED MALATE TRANSPORTER 1 (ALMT1). Malate excreted by ALMT1 chelates Al^3+^ ions and prevents its entry into the cell (Hoekenga et al., 2006; Iuchi et al., 2007), thus conferring Al tolerance to the root. Organic acid exudation is, in fact, the best understood Al-exclusion mechanism in plants, and is present in several plant species (Kochian et al., 2015). Further research demonstrated that STOP1 also regulates the expression of several genes involved in ion homeostasis and metabolic pathways that also contribute to Al tolerance such as the citrate transporter *MULTIDRUG AND TOXIC EXTRUSION (MATE1)* and the *ALUMINUM SENSITIVE3* (*ALS3)* a gene that codes for an ABC-like transporter protein and whose mutant (*als3*) is also hypersensitive to Al toxicity (Larsen et al., 2005; Liu et al., 2009; Sawaki et al., 2009).

Besides proton and Al toxicity, another limiting factor for plant growth in in acidic soils is low Pi availability. Under acidic conditions, Pi is rapidly fixed by Al and Fe cations making it unavailable for plant uptake. The responses of plants to low Pi availability have been studied thoroughly (for review see López-Arredondo et al., 2014) and include systemic responses to optimize internal Pi homeostasis and morphological adaptations of the root system to enhance Pi scavenging from upper soil layers where Pi tends to accumulate. Root morphological adaptations in Arabidopsis include an increase in the density and size of root hairs, an increase in lateral root number and the inhibition of primary root growth (Péret et al., 2011). Two recent genetic screenings of EMS-mutagenized seedlings identified a role for STOP1 in the inhibition of root growth in response to low Pi availability (Balzergue et al., 2017; Mora-Macías et al., 2017). These reports proposed that STOP1 activates *ALMT1* transcription under Pi-limiting conditions, leading to the adjustment of primary root growth through the activation of a reactive oxygen species (ROS) signaling pathway, triggered by the malate-dependent accumulation of Fe in the apoplast (for review see Abel, 2017). These reports suggest that organic acid exudation serves a triple role in acidic soils by preventing toxic Al from entering the cell, performing anion displacement to release Pi for plant uptake and enabling root modifications to more efficiently explore the topsoil. Further studies on the subject demonstrated a role for two other Al-tolerance related proteins, ALS3 and SENSITIVE TO ALUMINUM RHIZOTOXICITY (STAR1), in root developmental responses by modifying iron homeostasis in Arabidopsis (Dong et al., 2017). STOP1 regulates the expression of both *ALMT1* and *ALS3*, highlighting STOP1 as a major regulatory hub of responses to the conditions present in acidic soils including low Pi, high Fe availability and Al toxicity.

Given the multifunctional role of STOP1 under acidic soil conditions, a question that arises is: How is the activity of the transcription factor regulated in response to multiple stress factors? Earliest evidence suggested that, because the transcription levels of *STOP1* do not significantly change in response to low pH or Al exposure, STOP1 was post-transcriptionally activated (Sawaki et al., 2009). A recent report on STOP1 regulation corroborated that the transcription factor is regulated at the posttranslational level *via* protein accumulation/stabilization under low Pi and low pH conditions when Fe and Al are present in the medium (Godon et al., 2019). Furthermore, it was demonstrated that STOP1 abundance is regulated by the ubiquitin-proteasome-mediated degradation pathway *via* a member of the F-box E3-type ubiquitin ligase protein family, REGULATION OF ALMT1 EXPRESSION (RAE1). This F-box protein directly binds STOP1 and lack of a functional RAE1 leads to higher levels of STOP1, with the concomitant upregulation of *ALMT1* (Zhang et al., 2019). Because STOP1 regulates *RAE1* expression, authors concluded that STOP1 autoregulates its turnover by upregulating *RAE1* expression and, therefore, there must be another interacting partner that triggers an initial accumulation of STOP1. A dissection of how STOP1-targets are regulated in response to single and combinatorial stress conditions may provide insights into the mechanism(s) that modulate STOP1 activity.

Because of the overlap in the processes that are activated in response to the different stress conditions present in acidic soils, it remains unclear which responses are either shared by or specific to each type of stress. Extensive transcriptional profiling of the response to low Pi availability (Misson et al., 2005; Thibaud et al., 2010; Hoehenwarter et al., 2016; Mora-Macías et al., 2017), Al^3+^ toxicity (Sawaki et al., 2009; Kusunoki et al., 2017) and low pH (Sawaki et al., 2009; Lager et al., 2010) has been performed, however, a combinatorial study that dissects the specificity of the responses is lacking. Analysis of *stop1* global transcriptional changes in response to combinatorial stress conditions could provide insights into the STOP1-dependent regulation of genes because it would elucidate whether STOP1 activates the expression of the same gene set or specific gene sets in response to low Pi, low pH or combined Al^3+^ and Fe^3+^ stress conditions. Transcriptional profiling of *stop1* mutants could also provide insights into STOP1 dependent and independent mechanisms underlying tolerance to proton and metal toxicity. A transcriptomic characterization of *stop1* mutants in response to some of the individual stresses prevalent in acidic soils was previously reported (Sawaki et al., 2009), nonetheless, in this previous study microarray technology was used, which has limited dynamic range when compared to modern RNA-sequencing technology.

In this study, we perform a dissection of the transcriptional responses that are activated by the roots of wild-type and *stop1* Arabidopsis seedlings when exposed to factors that affect plant growth in acidic soils, namely, low Pi availability, low pH, Fe excess and Al toxicity, using RNA-sequencing technology. Our data suggest that a large portion of the transcriptional response is shared by multiple stress conditions, nonetheless, there are specific subsets of genes that are activated only in response to specific stress conditions. We also report that the expression of some STOP1-target genes correlates with the accumulation of STOP1 in the nucleus, whereas others do not follow this trend. We provide this dataset to the community with the intention of moving the field forward by accelerating the identification of new candidate genes that regulate root tolerance to acidic soil conditions.

## Materials and Methods

### Plant Material

*Arabidopsis thaliana* (Col-0 ecotype; CS70000) seeds were used as the wild-type genotype in this study. *stop1-ko* T-DNA line SALK_114108 was used as the *stop1* (Col-0 ecotype background) mutant genotype.

### Gene Cloning and Plant Transformation

The *STOP1* gene (AT1G34370) was cloned using the Golden Gate (GG) Strategy (Engler and Marillonnet, 2014) to produce a scar-free translational fusion to mCherry, as depicted in **Supplementary Figure 1**. We cloned the *STOP1* promoter (proSTOP1; 2085 bp upstream of the 5’ Untranslated Region (UTR) of the *STOP1* gene), the 5’UTR (556 bp), 3’UTR (128 bp) sites and the *STOP1-CDS* sequence (1497 bp) into L0 vectors from the GG Plant Toolkit (Engler et al., 2014). Then we performed the L1 synthesis reaction as instructed in Engler and Marillonnet (2014) and added the *mCherry* gene previously cloned in L0 vector that comes readily available in the GG Plant Toolkit, to produce the final synthesis of the C-terminal fusion of mCherry and STOP1 with the STOP1 promoter sequence and native UTR sites (proSTOP1::5’UTR::STOP1~mCherry::3’UTR referred as proSTOP1::STOP1::mCherry in this text for simplicity). proSTOP1::STOP1::mCherry was cloned into an L2 GG binary vector that we introduced into *Agrobacterium tumefaciens* by electroporation. Agrobacterium tumefaciens containing proSTOP1::STOP1::mCherry was used to transform of *stop1* plants using the floral dip method as described in Martinez-Trujillo et al., 2004. Out of 10 transgenic lines that complemented the *stop1* mutant phenotype (under low Pi and low pH conditions) two single locus, homozygous proSTOP1::STOP1~mCherry, lines without any apparent abnormal phenotypes, were selected for further characterization. Primers used to clone the *STOP1* L0 modules are the following: *proSTOP1* (forward (fw): 5’-ttgaagacaaggag gatttcgcgaatccgaat-3’; reverse (rv): ’-ttgaagacaaagtaggggtgctctccactttc -3’), 5’UTR (fw: 5’-ttgaagacaatactaaagctaataaacatgagccc-3’; rv: 5’-ttgaagacaacattttttagttcaagatcttgtttttc-3’), *STOP1-CDS* (fw: 5’- ttgaagacaaaatggaaactgaagccgatttgtg -3’; rv: 5’- ttgaagacaacgaagcaatgcctttgagactagtatc -3’) and 3’-UTR (fw:5’-ttgaagacaagctt ggcattgccatatatatgataag-3’; rv:5’-ttgaagacaaagcgaagaaccaatctttctgctattc-3’).

### Complementation Test

**Figure 1:**
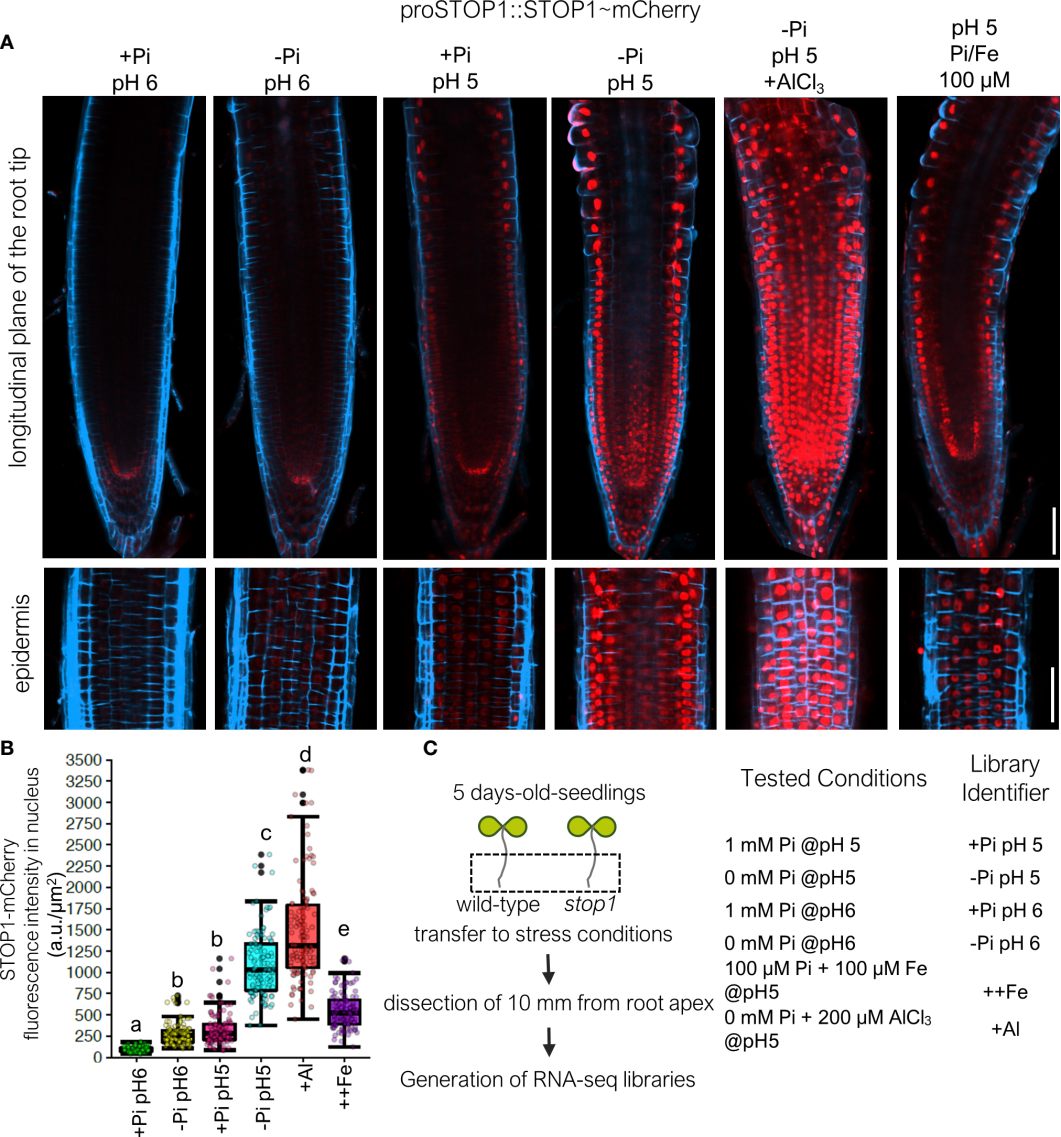
STOP1 accumulation as a molecular marker to direct RNA-seq profiling of acidic pH, low Pi, Al toxicity and Fe toxicity conditions. **(A)** Transgenic *stop1* seedlings expressing proSTOP1::STOP1~mCherry (line #1) were grown 5 days-after-germination (dag) and then transferred to the indicated medium conditions during 16h, at this time STOP1-mCherry signal (Red Channel) was observed using confocal microscopy. The cell-wall was stained using a modified DAPI-staining (see Materials and Methods). Upper panels depict a longitudinal plane of the root apex; lower panels depict epidermis layer of the root apex. Scale bar equals 50 µM. **(B)** Quantification of STOP1-mCherry signal in the nucleus of epidermal cells under the tested conditions in 2 biological replicates using 2 independent transgenic *stop1* seedlings expressing proSTOP1::STOP1~mCherry with 3 technical replicates. A total of n=112 nuclei per condition were measured. Statistical groups are represented by letters and were determined using a Tukey HSD test (P-val <.05). **(C)** RNA-seq profiling experimental strategy and design.

For complementation experiments (**Figure 1** and **Supplementary Figure 1**) we surface sterilized seeds and sowed them in 1% agar and 10% Murashige and Skoog Medium as described in López-Bucio et al. (2002). Low Pi medium (-Pi) was prepared with a concentration of 0 mM KH_2_PO_4_ and high Pi (+Pi) medium was prepared using 1 mM KH_2_PO_4_; sucrose concentration was 1% and MES at a 3.5 mM concentration to buffer pH Medium. MES optimum buffer range is 5.5 - 6.7, however, we added MES to keep pH below 5.5 which is already toxic to plants and is suitable for testing Al toxicity (Kobayashi et al., 2013). Medium was prepared at high Pi or low Pi concentration with pH adjusted to pH 5 or pH 6 as indicated in the text and figures. Wild-type plants (used as control) and *stop1* seedlings were grown for 10 days after germination (dag) in a Percival chamber at 22°C, under 16/8 h photoperiod with >200 μmol·m^2^·s^1^ photon flux density.

### Preparation of Root RNA-Seq Libraries

For the preparation of RNA-seq libraries, plant seedlings were germinated in high Pi medium at pH 5.7 as described in the previous section and 5 days after germination seedlings were transferred to the specified treatments under hydroponic conditions (no agar was added to the medium; 4 mL of each specific medium were added to 6-well cell culture Corning plates) specified in **Figure 1C** during 16 h (Percival chamber at 22°C, 8/8 h photoperiod with >200 μmol·m^2^·s^1^ luminous intensity). Base medium for the preparations of specific treatments was the same as described in complementation studies with the exception that agar was not added to the medium. For the case of +Al-treatment, Aluminum was added as AlCl_3_ at a concentration of 200 µM. For the case of ++Fe (Fe excess) treatment Fe was added in as FeSO_4_ to a final concentration of 100 µM and KH_2_PO_4_ was added to achieve a final concentration of a 100 µM Pi. Given the lack of agreement between exposure times in the literature (Sawaki et al., 2009; Lager et al., 2010; Zhang et al., 2019) which ranged from 1-24 h of exposure to stress treatment we decided to use 16h to ensure that STOP1 was active. It is possible that 16h exposure to low pH, Al and Fe treatments induced ROS production and some degree of cellular damage because of the relatively long exposure to the stress, however, our treatment was within the time of exposure that has been tested previously which is generally within the 24h range (Sawaki et al., 2009; Zhang et al., 2019). In fact, Arabidopsis seedlings can survive for up to 7 days in low pH medium with an aluminum concentration in the 200-500 μM range (Hoekenga et al., 2006; Illéš et al., 2006). Because we also observed differential STOP1-mCherry accumulation in the root in response to tested treatments (**Figure 1**), we concluded that our exposure time (16h) was adequate for transcriptional profiling. After the 16 h treatments, frozen root tip powder was obtained from root tip sections of approximately 10 mm in length from approximately 150 individuals per treatment. Total RNA was isolated using TRIzol (Invitrogen) from frozen root powder obtained from two independent biological replicates for each treatment reagent. Strand-specific mRNA-seq libraries were generated using the TrueSeq Illumina protocol and sequenced using the Illumina platform (paired-end reads, 150 base pairs; HiSeq2500). We calculated free Fe-availability in the medium using the chemical speciation software GEOCHEM-EZ (Shaff et al., 2010).

### Confocal Microscopy and Fluorescence Signal Quantification

Roots were harvested and mounted after the specified treatments in **Figure 1**. Root cell-wall was stained using a modified DAPI staining. DAPI staining solution was prepared at this time at 0.1 µg/µL in the respective liquid culture medium of the tested conditions (see **Figure 1C**). The roots of proSTOP1::STOP1~mCherry seedlings were mounted on the DAPI-staining solution followed by incubation for 5 min and then imaged with a Zeiss LSM800 upright confocal microscope using a 405 nm Laser line (for DAPI) and a 561 nm Laser line (for mCherry). Fluorescence signal quantification was performed using FIJI software [version 2.0; (Schindelin et al., 2012)] using a protocol by Luke Hammond available on GitHub https://github.com/mfitzp/theolb/blob/master/imaging/measuring-cell-fluorescence-using-imagej.rst; scale was calibrated to pixels/µm and mean fluorescence intensity/µm value was used.

### Determination of Number of Replicates per Sample for Bioinformatic Analysis

Biological variation is an important parameter to consider in RNA-seq protocols, hence the need to perform biological replication. To determine whether the level of biological replication in our RNA-seq analysis was adequate, we performed an analysis of the biological coefficient of variation (BCV), defined in edgeR (Robinson et al., 2010) as a parameter to account for variation between biological replicate libraries. According to the edgeR manual (http://bioconductor.org/packages/release/bioc/vignettes/edgeR/inst/doc/edgeRUsersGuide.pdf), a reasonable BCV value is less than 0.4 for a well-controlled experiment with adequate biological replication. Using edgeR, we determined that the BCV value of the biological replicates in our study is 0.1612 (**Supplementary Figure 4**), which is acceptable within edgeR standards and provides a statistical framework for determining significant differential expression between contrasting treatments. To test the levels of astringency that we were using, we decided to use a suggested methodology in the edgeR manual that is useful when there is no biological replication and that consists in selecting “housekeeping” genes, genes that do not variate in response to the tested treatments and have a relatively high level of expression, and calculate the BCV of these genes assuming a similar set of libraries as replicates. In this case, we assumed all wild-type and all *stop1* libraries, respectively, as replicates resulting in 12 replicates per genotype. The BCV of 100 housekeeping genes selected from our data set (included in **Supplementary Table 1**) resulted in a BCV value of 0.06767, less than 2 times the actual BCV of the study, indicating that our approach was at least two times more astringent than the housekeeping approach. This result corroborated that we could proceed with our analysis of differential expression with statistical certainty and that two replicates per sample was an adequate number for the purpose of the analysis reported in this work.

### Bioinformatic Analysis of RNA-Seq Data

We performed quality assessment of the resulting reads from the Illumina platform using FastQC (version 0.11.9; https://www.bioinformatics.babraham.ac.uk/projects/fastqc/) and processed sequencing libraries using Trimmomatic (version 0.39; (Bolger et al., 2014)) to remove adapter read sequences. Paired-end reads were aligned to the Arabidopsis genome (TAIR10; Release 46) using HISAT2 (version 2.1.0; (Kim et al., 2015)). Raw counts of read alignment per gene/locus were calculated using HTSeq (version 0.11.2; (Anders et al., 2015)). Differential expression analysis was carried out in R using edgeR package (version 3.28.0; (Robinson et al., 2010)) available from Bioconductor site (http://bioconductor.org/). Gene expression is represented by the normalized raw counts per gene (edgeR’s counts per million reads (cpm)). Cpm are obtained as raw counts per gene and normalized by library size. Heatmaps and graphs of the behavior of expression were represented using the z-score ((expression value in cpm – mean cpm across all the conditions tested)/standard deviation of gene expression in cpm across all the conditions tested). Pairwise comparisons were performed using edgeR’s glmLRT function, resulting changes were represented using the log2 of fold change (logFC). Venn analysis was performed in R using UpSet package (version 1.4; (Lex et al., 2014)). Gene Ontology (GO) analysis was carried out using the Classification Superviewer from the Bio-Analytic Resource for Plant Biology at http://bar.utoronto.ca/ntools/cgi-bin/ntools_classification_superviewer.cgi (Provart and Zhu, 2003), a summary of the output is presented in **Figure 4**, complete GO analysis with GO identifiers is included in **Supplementary Table 1**.

## Results

### STOP1 Accumulation as a Molecular Marker to Direct RNA-Seq Profiling

Since STOP1 has been reported to accumulate in the nucleus of epidermal root cells in response to several stress factors present in acid soils (Godon et al., 2019), we decided to test whether the accumulation of STOP1 could be used as a marker to determine the level of stress and/or transcriptional responses of the Arabidopsis root to different factors. With this aim, we generated a translational fusion of STOP1 to a fluorescent protein (mCherry) to use STOP1 accumulation as a stress marker to guide transcriptomic profiling (**Figure 1**). To confirm that the STOP1-mCherry fusion was functional in a biological context, we transformed a *stop1* mutant (Col-0 ecotype) with a construct that expresses a STOP1-mCherry fusion protein under the control of the endogenous *STOP1* promoter (proSTOP1::STOP1~mCherry; **Supplementary Figure 1A**) and tested for complementation. We isolated two independent transgenic lines with single locus insertion in which STOP1-mCherry was detected in the nucleus of root cells (**Figure 1A**; **Supplementary Figure 1D**) and that complemented the *stop1* mutant phenotype under low pH and low Pi with no apparent phenotypes other than a slight, but statistically significant, root hypersensitivity to low Pi (**Supplementary Figures 1B, C**). Because the proSTOP1::STOP1~mCherry construct was able to complement the *stop1* mutant phenotype under low pH and low Pi conditions, respectively, and because we observed differential accumulation of STOP1~mCherry in response to low Pi and low pH as has been previously reported for STOP1 (Balzergue et al., 2017; Godon et al., 2019) we concluded that the STOP1~mCherry fusion is functional in a biological context. Then, we decided to monitor STOP1-mCherry accumulation in the roots of seedlings exposed for 16h to low Pi conditions (0 mM Pi, pH 6), low pH conditions (1 mM Pi, pH 5), low pH and low Pi conditions (0 mM Pi, pH 5), Al toxicity (0 mM Pi, 200 μM AlCl_3_, pH 5) and Fe excess (100 μM Pi, 100 µM Fe, pH 5) and compared it to that observed under control conditions (1 mM Pi, pH 6). We observed that STOP1 accumulates differentially in the root tip (**Figure 1A** upper panel), in response to all the conditions tested and that these differences were most evident in epidermal cells (**Figure 1A**, lower panel). STOP1 accumulated in response to individual low pH and low Pi treatments, nonetheless, the effect was potentiated up to 4 orders of magnitude when both treatments are combined (**Figure 1B**). As expected, maximum accumulation of STOP1 was observed in Al-treatment which has a combination of stress treatments including low Pi, low pH and Al presence (**Figures 1A, B**). To simulate increased Fe availability conditions similar to those that happen in acidic soils, at pH 5 we increased Fe supply 10 times and decreased Pi supply 10 times (100 µM supply of Pi and Fe) relative to control conditions (1 mM Pi, 10 µM Fe). We observed that Fe excess triggered accumulation of STOP1 at high levels, however, not as high as those observed for seedlings exposed to low Pi at pH 5 or Al-treatment (**Figure 1B**). This result may indicate that Al has a stronger effect than Fe on the accumulation of STOP1, however, we cannot rule out a low Pi effect on the accumulation of STOP1 in the Al treatment because the Pi concentrations in those treatments was lower than in the Fe treatment (0 μM Pi in Al treatment; 100 μM Pi in Fe excess treatment). We determined that the observed differences in STOP1 accumulation were statistically significant by quantifying the intensity of the STOP1-mCherry signal in the nucleus of epidermal cells from root tips exposed to low Pi, low pH, Al and Fe excess at low pH (**Figure 1B**). It was recently reported that a relative increase of Fe in low Pi media triggers STOP1 accumulation under low pH conditions (Godon et al., 2019). Our data agrees with this report, because we also observed that STOP1 accumulation increases under low pH and elevated Fe levels in the medium. However, our data suggests that low Pi at low pH alone has a greater effect than that of Fe-excess in the accumulation of STOP1, therefore, Pi availability has a more determinant effect on STOP1 accumulation than Fe-excess. We cannot rule out that this effect is due to a modification of the Pi/Fe ratio and that a Fe-threshold in the medium may be sufficient for STOP1 accumulation. This last possibility is unlikely because in the low Pi media the Fe concentration is 10 μM, with a calculated free Fe availability of 50.5% (see Materials and Methods), which is much lower than the Fe concentration (100 μM) and calculated free Fe availability (86.97%) of the Fe-excess treatment.

Since we observed nuclear accumulation of STOP1 under all our proposed treatments (**Figure 1B**), we decided to perform RNA-seq profiling in the roots of wild-type (Col-0 ecotype) and *stop1* seedlings (Col-0 ecotype) that were exposed to low pH, low Pi, Al and Fe-excess treatments which simulate the conditions present in acidic soils *in vitro* (**Figure 1C**).

### Multiple Subsets of Genes Are Differentially Regulated in Response to Low Pi, Low pH, Al-Exposure and Fe-Excess

**Figure 2:**
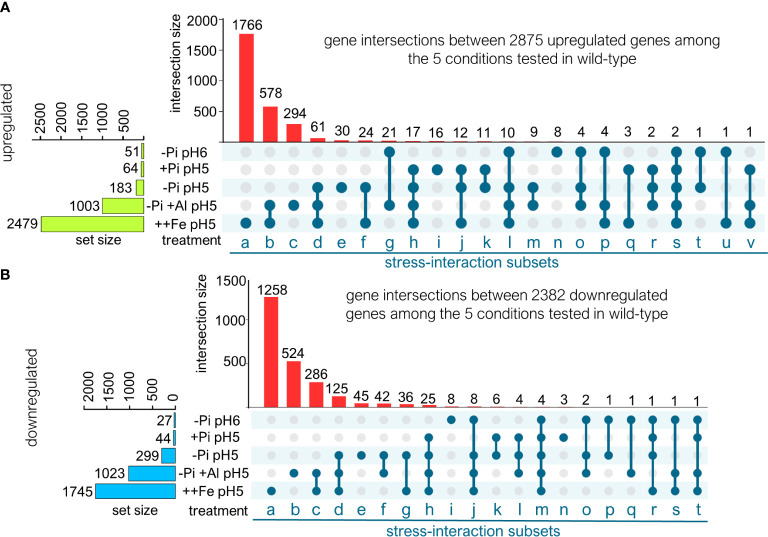
Transcriptomic dissection of upregulated and downregulated gene expression in response to low pH, low Pi, Al and Fe excess. **(A)** Venn analysis of genes that are differentially upregulated (FDR <.05) under the conditions tested in wild-type (using wild-type pH 6 high-Pi as baseline). Venn diagram is represented in UpSet plot style (see Materials and Methods). Resulting intersection subsets are named using letters. **(B)** Venn analysis of genes that are differentially downregulated (FDR <.05) under the conditions tested in wild-type (using wild-type pH 6 high-Pi as baseline). Venn diagram is represented in UpSet plot style (see Materials and Methods). Resulting subsets are named using letters.

For RNA-Seq analysis, seedlings were exposed for 16h to low Pi conditions (0 mM Pi, pH 6), low pH conditions (1 mM Pi, pH 5), low pH and low Pi conditions (0 mM Pi, pH 5), Al toxicity (0 mM Pi, 200 μM AlCl_3_, pH 5) and Fe excess (100 μM Pi, 100 µM Fe, pH 5) and RNA was extracted from root tissue. We selected this time point because it was previously reported that the majority of STOP1-dependent genes are not activated at early time points, shorter than 8h, in response to low pH stress (Lager et al., 2010). Strand-specific RNA-Seq libraries for two independent biological replicates for each treatment were prepared using polyA+ RNA and sequenced using an Illumina HiSeq platform. A summary of the reads obtained for each library and alignment percentage is presented in **Supplementary Table 1**. Once RNA-sequencing was performed we decided to perform pairwise comparisons of the treatments (-Pi_pH6, +Pi_pH5, -Pi_pH5, +Al, ++Fe) with respect to control conditions (+Pi_pH6) to determine the genes that are differentially expressed (false discovery rate <.05) in response to each treatment (**Figure 2**, **Supplementary Table 1**). We determined that 51 and 27 genes were upregulated and downregulated, respectively, in response to -Pi_pH6; 64 and 44 genes were upregulated and downregulated, respectively, in response to +Pi_pH5 treatment; 183 and 299 were upregulated and downregulated, respectively, in response to -Pi_pH5 treatment; 1003 and 1023 were upregulated and downregulated, respectively, in response to +Al treatment and, lastly, 2479 and 1745 were upregulated and downregulated, respectively, in response to ++Fe treatment. These data indicate that ++Fe treatment induces changes in the expression of the greatest number of genes followed by +Al, -Pi_pH5, +Pi_pH5 and -Pi_pH6 treatments in descending order. As the increase in STOP1 in the high Fe treatment was lower than the treatment with -Pi_pH5 and +Al, this fact suggests that a large portion of the transcriptional effect of high Fe treatment is independent of STOP1 and probably mediated by other transcription factors and signaling pathways.

We then decided to perform a dissection of the genes whose upregulation is shared or specific to each treatment (**Figure 2**). Using a Venn analysis approach, we determined 17 intersections between the upregulated genes among the five tested treatments, 8 of the intersections are larger than 10 genes and 3 share more than 50 in common genes (**Figure 2A**), indicating that there is a considerable portion of the transcriptomic response shared between two or more treatments. To further sustain this last conclusion, we calculated the percentages of specificity of upregulated genes for each treatment by dividing the number of genes that are not shared with other treatments by the number of genes that are upregulated by that treatment. The percentages of specificity are listed as follows: -Pi_pH6 (15.6%), +Pi_pH5 (25%), -Pi_pH5 (16.3%), +Al (29.3%) and ++Fe (71.2%). The percentages of specificity of each response indicate that, in low Pi, low pH and Al exposure treatments, over 70% of the transcriptional upregulation is shared by the three conditions. In the case of Fe-excess treatment, which induces the expression of the largest set of genes, 70% of upregulated genes are not shared with the other treatments and seem to be part of a specific response to elevated concentrations of Fe.

We continued our dissection of transcriptional responses by performing Venn analysis of intersections between downregulated genes. We found that the downregulation of genes in response to the tested treatments has less intersections between treatments. Fifteen intersection gene subsets were identified, which is two less than in the upregulation response (**Figure 2B**). At first, this number could indicate that downregulated genes are less stress-specific than upregulated ones. However, the number of specific genes that are downregulated indicates that this might not be the case because the percentages of specificity, calculated by dividing the number of genes downregulated in response to the treatment that are not shared with other treatments by the total number of genes downregulated by the treatment, are as follows: -Pi_pH6 (29.6%), +Pi_pH5 (6.8%), -Pi_pH5 (15%), +Al (51.2%) and ++Fe (72.1%). Overall, it seems that the downregulation of expression is more treatment-specific for -Pi_pH6, +Al and ++Fe. The finding that there are less interaction subsets and more specificity responses indicates that one or more downregulated interaction subsets are larger than the intersection subsets of upregulated genes. This is the case of the downregulated “subset d” which doubles its size (125 genes) with respect to upregulated genes (61 genes) and contains genes whose expression is coordinately regulated under low Pi, +Al, ++Fe at pH 5 conditions. This indicates that the root downregulates a common set of genes, larger than the one it upregulates, when exposed to low Pi, low pH, Al-exposure and Fe-excess. The complete dissection with gene identifiers for downregulated and upregulated responses is included in **Supplementary Table 1**.

### STOP1 Has a Major Influence on the Transcriptomic Landscape in Response to Low Pi, Low pH, and Al-Exposure

**Figure 3:**
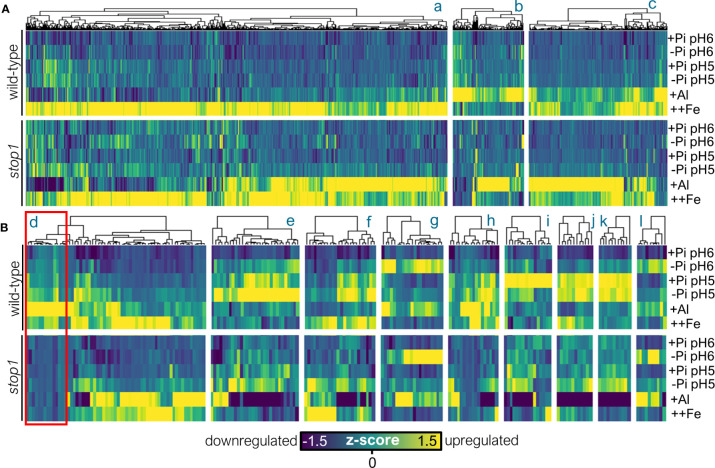
STOP1 has a major effect on the transcriptome landscape in response to low Pi, low pH and Al exposure conditions. **(A)** Upper panel: heatmap of normalized gene expression (z-score) in the roots of wild-type seedlings exposed to the indicated treatment. Differentially upregulated genes (FDR <.05) from upregulated subsets a-c (obtained from **Figure 2**) are presented and clustered using the Pearson method. Lower panel: heatmap of normalized gene expression (z-score) in the roots of *stop1* seedlings exposed to the indicated treatment. Differentially upregulated genes (FDR <.05) from upregulated subsets a-c (obtained from **Figure 2**) are presented and clustered using the Pearson method. The subsets a-c were obtained from the Venn analysis presented in **Figure 2A**, these are the stress-interaction or stress-specific subsets of genes upregulated in response to the tested treatments and have the same letter identifiers as in **Figure 2A**. **(B)** Upper panel: heatmap of normalized gene expression (z-score) in the roots of wild-type seedlings exposed to the indicated treatment. Differentially upregulated genes (FDR <.05) from upregulated subsets d-l (from **Figure 2**) are presented and clustered using the Pearson method. Lower panel: heatmap of normalized gene expression (z-score) in the roots of *stop1* seedlings exposed to the indicated treatment. Differentially upregulated genes (FDR <.05) from upregulated subsets d-l (obtained from **Figure 2**) are presented and clustered using the Pearson method. The subsets d-l were obtained from the Venn analysis presented in **Figure 2A**, these are the stress-interaction or stress-specific subsets of genes upregulated in response to the tested treatments and have the same letter identifiers as in **Figure 2A**.

Once we dissected the transcriptomic responses in the wild-type, we sought to analyze the effect of the *stop1* mutation on the regulation of transcription in response to the tested conditions (**Figure 3**). To gain further insights into the transcriptomic landscape of the upregulated response for each treatment, we generated heatmaps of the intersection subsets of more than 10 genes for both WT and *stop1* genotypes (**Figure 3**). Overall, we found that the response in *stop1* as compared to the wild-type, was mixed: 1) genes whose expression is downregulated in *stop1* with respect to the WT, which was visibly the major trend across all subsets (activated in the WT but not in *stop1*) and 2) genes whose expression is upregulated in *stop1* with respect to wild-type (**Figures 3A, B**). The finding that the expression of genes that belong to both stress-specific and shared subsets is downregulated in *stop1* seedlings (**Figures 3A, B**) is of major relevance because it indicates that the increased expression of these genes depends not only on the accumulation of STOP1 but also of other factors that are only activated by stress specific signaling pathways. Genes that are induced under all stress treatments and expression levels of which are dependent on the level of STOP1 accumulation, probably only require STOP1 to activate their transcription. In the case of genes that are stress-specific, the accumulation of STOP1 is insufficient to trigger expression, therefore, either other transcription factors activated by the specific stress are required to activate the expression of target genes or different post-translationally modified versions of STOP1 might exist that can differentially bind to the promoter sequence of the target genes in a stress-specific or more general manner.

The second major effect of the *stop1* mutation on the transcriptomic landscape in response to the tested treatments was the upregulation of genes with respect to wild-type, or in other words, an enhanced induction of genes in the roots of *stop1* seedlings in response to the tested stress conditions. This type of response was most evident in the case of subsets “b” and “c” which contain genes that are upregulated specifically by +Al treatment in which the expression of some genes is visibly more activated in *stop1* than in wild-type (**Figure 3A**). We reasoned that this type of transcriptional response could be qualified as a hypersensitive transcriptional response of *stop1* to Al-toxicity. It is most likely that, under the Al treatment conditions that we tested, malate-independent mechanisms to ameliorate Al toxicity were activated in *stop1* mutants because they are defective in malate excretion. In the case of subset “a” which is specific to ++Fe treatment, the transcriptional response was similar in *stop1* and the WT, indicating that STOP1 does not play a specific role in the regulation of transcriptional responses specific to ++Fe excess.

To investigate a possible hypersensitive transcriptional response to +Al treatment in *stop1*, we determined the number of differentially expressed genes in the roots of *stop1* exposed +Al with respect to control conditions (+Pi_pH6, WT). We found that in *stop1* 3431 genes were upregulated and 3680 downregulated, accounting for 3.5 times more differentially expressed genes in the *stop1* mutant than the WT. Then, we compared upregulated genes in STOP1 and the WT (**Supplementary Figure 2**). We observed that 758 genes are upregulated in both WT and *stop1*, whereas 246 genes are upregulated only in the WT and 2673 genes are upregulated only in *stop1* (**Supplementary Figure 2**).

To further understand the hypersensitive transcriptional response of *stop1* to Al-treatment we evaluated the two plant Al-tolerance responses that have been previously described: Al-exclusion, which focuses on preventing Al-entrance to the cell, and Al-detoxification, which focuses on detoxifying the cell once Al has crossed inside the plasma membrane (Kochian et al., 2015). We hypothesized that the hypersensitive transcriptional response to Al-treatment that is triggered in *stop1* mutants occurs because these mutants are defective in Al-exclusion which then leads to a hyper-activation of Al-detoxification. As expected, the expression of *ALMT1, MATE1, PGIP1, ALS3* that participate in preventing the entry of Al into root cell is only activated in wild-type and not in *stop1*. This was not a surprise because these genes have been previously reported to be downregulated in *stop1* mutants (Sawaki et al., 2009). We then analyzed the list of genes that are activated in response to +Al-treatment in *stop1* but not in WT and found genes coding for glutathione-S-transferases (*AT1G69920, AT1G10370, AT2G02380, AT2G47730*) which have been proposed to be involved in detoxifying Al-generated ROS (Daspute et al., 2017), a gene that codes for the tonoplast transporter *ALUMINUM SENSITIVE 1* (*ALS1; AT5G39040*) that contributes to Al-detoxification by sequestrating Al into the vacuole (Larsen et al., 2007; Huang et al., 2012) and *MONODEHYDROASCORBATE REDUCTASE* (*MDAR1; AT3G52880)* a gene whose product is related with hydrogen peroxide detoxification (Daspute et al., 2017). These results further support the hypothesis that Al-detoxification is induced in the root when Al-exclusion is defective or insufficient to prevent the entry of Al into the root.

We observed that the transcriptional response to ++Fe treatment in *stop1* was the least affected of all the conditions tested (**Figure 2**), but also that a portion of Fe-responsive genes were hyper-activated in the roots of Al-treated *stop1* seedlings. This indicates that there are detoxification mechanisms that contribute to both Al and Fe tolerance and are more active when STOP1 is missing most likely because Al- and Fe-exclusion mechanisms are defective in *stop1*. By using hierarchical clusterization, we determined the set of Fe-responsive genes that are hyper-activated in the roots of Al-treated *stop1* seedlings (**Supplementary Table 1**). Among these genes, we found several genes related to metal and oxidative stress detoxification including *ASCORBATE PEROXIDASE 1 (APX1)* whose mutants are defective in H_2_O_2_-scavenging and in which cytosolic protein oxidation occurs (Davletova et al., 2005), and several genes coding for glutathione-S-transferases (*GST8, GST25, GST29, GSTL3)* that have been related with detoxification of Al-induced ROS (Daspute et al., 2017) and *DEHYDROASCORBATE REDUCTASE 2 (DHAR2)* a gene that is related with the modulation of cellular redox states under oxidative stress conditions (Noshi et al., 2017). Because Fe-excess and Al-toxicity can occur simultaneously in acidic soils and both metals are potent elicitors of oxidative stress, these genes represent interesting candidates that contribute to the detoxification of ROS in response to Fe/Al-induced oxidative stress. The full list of genes is included in **Supplementary Table 1**.

### Genes Encoding Proteins With Kinase Activity, Detoxification, Transport, Phosphate Starvation Response, and Cell Wall Related Processes Are Upregulated in Response to Low Pi, Low pH, and Al Treatment

**Figure 4:**
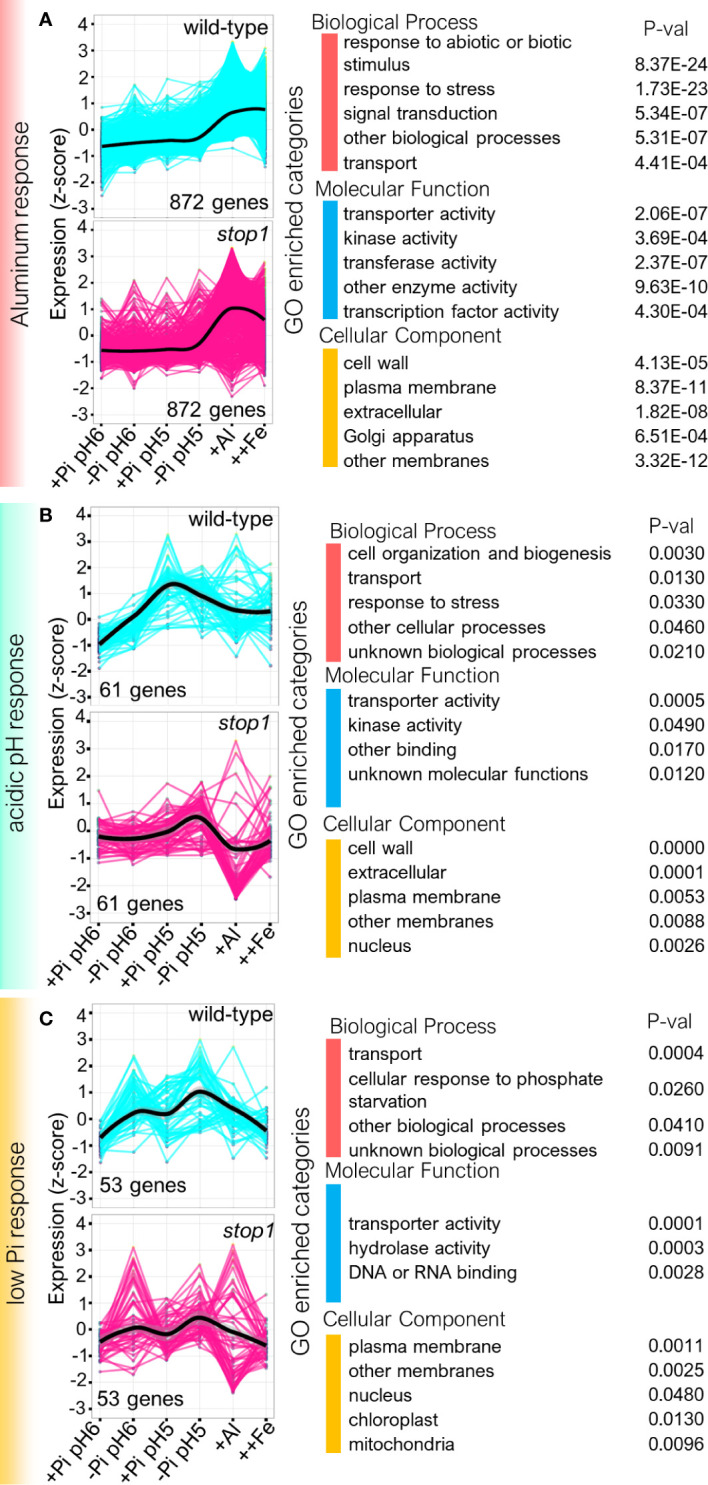
Expression profiling and Gene ontology (GO) analysis of the transcriptionally upregulated processes in the roots of wild-type and *stop1* seedlings in response to low Pi, low pH and Al. **(A)** GO profiling of genes whose expression is significantly induced in wild-type (FDR < 0.05) in response to Al or to Al and Fe treatment (subsets b + c from **Figure 2A**) this gene set is referred to in the text and the figure as the *Aluminum response*. Left: Normalized expression profiles (z-score; y-axis) of *Aluminum response* genes in the roots of wild-type and *stop1* seedlings in response to the tested treatments (x-axis). Right: Summary of GO analysis performed using the Classification SuperViewer with Bootstrap (see Materials and Methods). For the complete analysis including GO categories for each of the classes presented and gene identifiers see **Supplementary Table 1**. **(B)** GO profiling of genes whose expression is significantly induced in wild-type (FDR < 0.05) in response to pH 5 across all treatments (subsets h + i + j + k + q + r from **Figure 2A**), this gene set is referred to as the *acidic pH response*. Left: Normalized expression profiles of *acidic pH response* genes (z-score; y-axis) in the roots of wild-type and *stop1* seedlings in response to the tested treatments (x-axis). Right: Summary of GO analysis performed using the Classification SuperViewer with Bootstrap (see Materials and Methods). For the complete analysis including GO categories for each of the classes presented and gene identifiers see **Supplementary Table 1**. **(C)** GO profiling of genes whose expression is induced in low Pi across all treatments (subsets e + l + n + o + t from **Figure 2A**), this gene set is referred to in the text as *low Pi response*. Left: Normalized gene expression profiles of *low Pi response* (z-score; y-axis) in the roots of wild-type and *stop1* seedlings in response to the tested treatments (x-axis). Right: Summary of GO analysis performed using the Classification SuperViewer with Bootstrap (see Materials and Methods). Significantly enriched GO processes have a P-value <0.05. For the complete analysis including GO categories and gene identifiers see **Supplementary Table 1**.

Because low pH, low Pi and Al responses were the most transcriptionally affected in the roots of *stop1*, we sought to determine the genes that are activated in the wild-type in response to low Pi, low Pi and Al, to analyze their gene expression pattern in wild-type and *stop1* backgrounds and then perform a Gene Ontology (GO) enrichment analysis. With this aim, we first clustered gene subsets from our previous transcriptomic dissection by their specific response to low Pi, low pH or Al treatment. We named these subsets the *Aluminum response*, the *low pH response* and the *low Pi response* (**Figure 4**). In the case of the low pH response we analyzed the genes that respond to pH 5 across all treatments (subsets h + i + j + k + q + r), for the low Pi response we analyzed the genes that respond to low Pi across all treatments (subsets e + l + n + o + t) and for Aluminum response we analyzed the genes that respond to Al or to Al and Fe treatment (subsets b + c). We then carried out the GO enrichment analysis of the biological processes, molecular function and cellular components associated with the encoded proteins of the genes that belong to each transcriptional response subset. We present a summary of functional categories (**Figures 4A–C**) that were activated for each response, grouped by biological process, molecular function and cellular component, the complete GO analysis including category names and identifiers (**Supplementary Table 1**). Furthermore, to get a notion of the changes in expression of the genes involved in such response in *stop1* vs WT, we generated graphs of the behavior of expression of each gene under each specific condition for the two tested genotypes and fitted a trend-line of the overall behavior of gene expression (**Figures 4A–C**).

For the case of the response to acidic pH (**Figure 4B**) we found enriched categories in biological processes related to cell organization and biogenesis, transport and response to stress, GO enriched categories for this gene set included [GO:0048768] root hair cell tip growth, [GO:0042545] cell wall modification, [GO:0006810] transport and [GO:0006979] response to oxidative stress. Molecular functions related to transporter activity and kinase activity were also enriched in the set of genes that are responsive to acidic pH. Moreover, the genes that code for enzymes whose products are targeted to the cell wall and extracellular space were the most enriched cellular components in the low pH response. The gene expression graphic and trendline show that the response to low pH is severely downregulated in *stop1* mutants across all the treatments tested (**Figure 4B**). These data highlight a role for the genes that code for cell wall proteins, kinases and transport related processes in root acclimation to acidic conditions.

The GO analysis of the Aluminum response (**Figure 4A**) indicates that, overall, biological processes related to stress responses are activated in the root in response to Aluminum stress conditions, including categories like responses to hydrogen peroxide [GO:0042542], salt stress [GO:0009651] and oxidative stress [GO:0006979]. In the specific case of the response to hydrogen peroxide we found two genes coding for transcription factors that belong to the family of *HEAT SHOCK FACTOR (HSF)*, namely, *HSFA1E* and *HSFA3*. The HSF-family of transcription factors has been related to the response to a myriad of abiotic stresses including heat, drought, hypoxia and oxidative stress (Guo et al., 2016). The expression of *HSFA1E* has been reported to be upregulated in response to H_2_O_2_ treatment and a quadruple mutant of *HSFA1A/B/D/E* was reported to be more sensitive to H_2_O_2_ treatment than the wild-type (Liu et al., 2011) confirming a role of this subfamily of transcription factors in the response to hydrogen peroxide. In the case of *HSFA3*, it was demonstrated that overexpression of its transcriptional activator DREB2C (Chen et al., 2010), a transcription factor involved in the response to drought, upregulates *HSFA3* expression and confers tolerance oxidative stress (Hwang et al., 2012). Interestingly, we observed that *DREB2C* expression was also upregulated in response to Al treatment (**Supplementary Table 1**), suggesting an overlap in drought-activated responses and Al^3+^ stress. It is likely that these changes occur because both stresses lead to oxidative stress inside the cell. The *HSF* family of transcription factors activate the expression of *HEAT SHOCK PROTEINS* (*HSP)* which act as chaperones that regulate the folding, localization, accumulation and aggregation of proteins during stress conditions, including oxidative stress (Al-Whaibi, 2011). We found that 4 *HSP* genes (*HSP21, HSP70, AT1G52560, AT2G29500)* which also belong to the hydrogen peroxide response were upregulated in response to Al^3+^ treatment. It is likely that Heat Shock (HS) related proteins protect the cell from oxidative stress during Al toxicity, however, further experimentation with HSFs and HSPs is required to better understand the role of HS related proteins in the tolerance to Al^3+^ stress.

In the case of molecular processes activated in the Aluminum response gene set (**Figure 4A**), we found that the transcript levels of several proteins with kinase activity are increased by Al stress. The upregulation of genes that code for kinases agrees with previous works reporting the involvement of protein phosphorylation in the response of plants to Al^3+^ stress (Osawa and Matsumoto, 2001; Panda and Achary, 2014; Ligaba-Osena et al., 2017). Among the multiple genes coding for kinases enriched in the Aluminum response we found *PROLINE RICH LIKE EXTENSION KINASE 4* (*PERK4*) whose mutant, *perk4*, has reduced sensitivity to abscisic acid (ABA) including reduced inhibition of root growth in response to ABA treatment (Bai et al., 2009). *perk4* mutants have lower cytosolic concentration of Ca^2+^ which causes defects in Ca^2+^-mediated ABA signaling (Bai et al., 2009). Interestingly, we also found that the expression of another Ca^2+^-signaling kinase *CBL-INTERACTING KINASE 17* (*CIPK17)* was induced in response to Al. *CIPK17* has been reported to participate in a signaling module that controls Ca^2+^ influx and regulates ABA-signaling during stomatal closure (Song et al., 2018). Moreover, the expression of *CALCINEURIN B-LIKE PROTEIN 1 (CBL1)* was also induced in response to Al, *cbl1* mutants have increased sensitivity to Al^3+^ toxicity and have downregulated expression of *CIPK17* (Ligaba-Osena et al., 2017). CBL1-CIPK17 have been reported to interact *in vivo* (Kolukisaoglu et al., 2004). Our results agree with previous findings that *CBL1* has a role in the activation of Al-tolerance and suggest a role for Ca^2+^-signaling, ABA-signaling and CBL1-CIPK17 signaling in the Al^3+^-toxicity response. Interestingly, Ca^2+^-signaling has also been reported to be involved in the early response to low pH (Lager et al., 2010). Further experimentation is required to more clearly understand the role of these signaling processes in the root tolerance to Al-stress.

The most enriched biological processes in the response to low Pi (**Figure 4C**) were transport and cellular response to phosphate starvation, including GO categories like [GO:0055085] transmembrane transport and [GO:0016036] cellular response to phosphate starvation. Transporter activity, hydrolase activity and DNA/RNA binding were the most enriched molecular functions. With respect to the cellular components, the plasma membrane was the most enriched organelle in the response to low Pi. Most of the low Pi-specific response is downregulated in the roots of *stop1* seedlings (**Figure 4C**). An interesting observation is that Fe excess cannot trigger the expression of Pi-responsive genes suggesting that Fe is not the trigger for the root responses to low Pi. To corroborate this, we analyzed the expression changes in response to the tested treatments of four genes that are well known to be induced by low Pi, *SPX DOMAIN GENE 1 (SPX1), PHOSPHATE TRANSPORTER 1;4, (PHT1;4) PHOSPHOLIPASE D ZETA 2 (PLDZ2), GLYCEROPHOSPHODIESTER PHOSPHODIESTERASE (GDPD1)* and *SULFOQUINOVOSYLDIACYLGLYCEROL 1 (SQD1)* (**Supplementary Figure 3**). The analysis showed that the expression of these 4 genes was only upregulated by low Pi conditions at both pH 6 and pH 5 and that ++Fe treatment was unable to upregulate their expression to the same extent of Pi-limiting conditions. These results agree with a previous report in which it was demonstrated that Fe does not trigger *SPX1* expression under low Pi conditions (Godon et al., 2019).

### STOP1 Activates a Specific Set of Targets in Response to Low pH, Low Pi, and Al-Exposure

**Figure 5:**
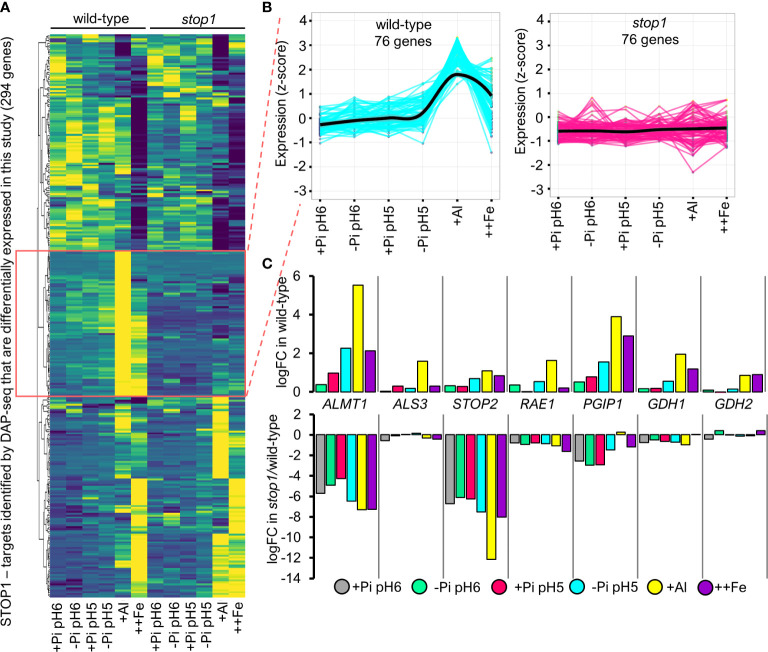
STOP1 triggers the expression of a specific set of its targets that correlate with its accumulation and are important for root tolerance in response to abiotic stress factors prevalent in acidic soils. **(A)** Heatmap of normalized gene expression (z-score) of 294 STOP1-target genes that are differentially expressed under any of the tested treatments (FDR <.05) identified by DNA Purification Affinity (O’Malley et al., 2016). Pearson clusterization was used to cluster genes with similar expression profiles, 5 gene subsets were obtained and the subset of 76 genes that correlate with STOP1 accumulation is highlighted. Gene identifiers and description of the 5 resulting subsets of STOP1-targets is included in **Supplementary Table 1**. **(B)** Normalized gene expression profiles (z-score; y-axis) of the subset of 76 STOP1-targets highlighted in **(A)** of wild-type and *stop1* seedlings in response to the tested treatments (x-axis). **(C)** Changes in the expression in log2 of fold change (logFC) of STOP1-targets in response to the indicated treatments with respect to the expression in control conditions (+Pi_pH6 treatment in wild-type). A table with the logFC of the 294 STOP1-targets is included in **Supplementary Table 1**.

Using DNA affinity purification sequencing technique (DAP-seq), a recent study reported a set of 1280 genes defined as STOP1-targets, because STOP1 binds their regulatory (promoter/cis) sequence (O’Malley et al., 2016). To determine which DEGs identified for the different treatments use in this study are direct targets of STOP1, we integrated the DAP-Seq data into our analysis (**Figure 5**). Out of 1280 STOP1-targets defined by DAP-Seq, 294 have differential expression in response to at least one of the conditions tested in our study. We then generated a heatmap to visually inspect the differences between the wild-type and *stop1* genotypes (**Figure 5A**). A subset of 76 co-expressed genes, marked in red in **Figure 5A**, showed upregulation in response to the tested treatments in the WT but not in s*top1* (**Figure 5A**). The effect of the *stop1* mutation in the expression of these genes is more evident when observed in a graph of expression vs treatment (**Figure 5B**). Moreover, the level of expression of these genes correlates with the increase in STOP1 accumulation (**Figure 1B**). Overall, these data suggest that this specific subset is integrated by genes that are direct targets of STOP1 whose expression is proportional to STOP1-accumulation. As the expression of these genes is upregulated by STOP1 in response to low Pi, low pH and Al-exposure, these genes apparently only require the accumulation of STOP1 and do not require of other factors stress-specific factors. Further support of this notion came from the fact that genes, such as *ALMT1, ALS3, STOP2, PGIP1, GDH1/2* (Iuchi et al., 2007; Sawaki et al., 2009) and *RAE1* (Zhang et al., 2019), for which experimental evidence shows that STOP1 binds to their promoter sequences are included in this subset. In the specific case of *ALMT1* and *RAE1*, it has been demonstrated both *in vitro* and *in vivo* that STOP1 binds to their promoter region (Tokizawa et al., 2015; Balzergue et al., 2017; Zhang et al., 2019).

The finding of STOP1-targets whose expression does not correlate with STOP1-accumulation suggests that some STOP1-targets require the presence of other transcription factors or interacting proteins to be activated. Two additional possible explanations for the finding of STOP1-targets that do not correlate with STOP1-acummulation is that STOP1 activates these additional targets in other tissues or in response to other types of stress that STOP1 responds to, including hypoxia and salt stress (Enomoto et al., 2019; Sadhukhan et al., 2019). However, because STOP1 regulation appears to be mainly post-translational (Godon et al., 2019; Zhang et al., 2019), differential activation of gene expression may also happen through a differential interaction with other transcription factors or regulating protein interactors. A fourth possible explanation is that some of these STOP1-targets are activated early in response to the treatments that we tested, and we were not able to detect them at the time that we harvested the tissue (16h) but in that case their expression would be mainly determined by the early presence of a transcription factor other than STOP1. Full list of STOP1-targets that are differentially expressed in our dataset is included in **Supplementary Table 1**.

## Discussion

### Transcriptional Profiling Provided Insights in the Regulation of Gene Expression in Response to Abiotic Stress Factors Prevalent in Acidic Soils

Acidic soils represent a challenge for modern agriculture, especially for developing countries of the tropical and subtropical areas of the world (von Uexküll and Mutert, 1995). In this work, we report a dissection of root transcriptional responses to the conditions present in acidic soil conditions, namely acidic pH, low Pi availability, Aluminum toxicity and Fe excess. We described an interesting subset of genes (**Figure 2**; subset d) for which expression is differentially regulated by all the factors that affect plant growth and development in acidic soils (low Pi, low pH, Al-toxicity and Fe-excess). This subset of genes includes genes previously demonstrated to play an important role in adaptation to low Pi, low pH and Al-tolerance such as *ALMT1* and *STOP2* (Kobayashi et al., 2014; Balzergue et al., 2017; Mora-Macías et al., 2017), but we also identified other novel genes for which induction is shared by all treatments and may serve as new marker genes for further studies of roots adaptation to acid soils. The fact that among this subset of shared genes, downregulated genes are twice more than upregulated genes (subset d; upregulated 61 genes, downregulated 125) suggests that the root might turn down the same cellular processes when it is exposed to any of the conditions present in acidic soils, whereas upregulated genes appear to be more stress-specific.

We observed that the transcriptional response to ++Fe treatment in *stop1* was the least affected of all the conditions tested (**Figure 2**), but also that a portion of Fe-responsive genes were hyper-activated in the roots of Al-treated *stop1* seedlings. This indicates that detoxification mechanisms that contribute to both Al and Fe tolerance are activated by mechanisms that are independent of STOP1. Nonetheless, our results demonstrate that *stop1* mutants are a suitable model to study the transcriptional activation of mechanisms related to Al-detoxification, and in some extent Fe-detoxification, because they are defective in organic-acid mediated exclusion of metals like Al and Fe. Genes that belong to the Al-detoxification set that are only activated in *stop1* represent interesting candidates to over-express to optimize Al-detoxification in plants. This last statement makes sense as Fe-excess and Al-toxicity are stresses that can occur together in acidic soils and both metals are potent elicitors of oxidative stress.

Analysis of GO enrichment in the sets of genes that are activated in response to low pH conditions (**Figure 4A**), revealed that genes that code for enzymes that are related to the modification of the cell wall are enriched in this gene set. Our results indicate that several genes related to pectin modification, a structural carbohydrate present in the root cell wall, including pectin methylesterase inhibitor genes (see **Supplementary Table 1**), are induced under low pH and Al^3+^ stress. Pectin methylesterase activity was positively correlated with sensitivity to Al^3+^ treatment in rice (Yang et al., 2013), suggesting that the induction of pectin methylesterase inhibitor genes might be a tolerance mechanism to Al^3+^ toxicity in plants. Furthermore, we found that the *POLYGALACTURONASE INHIBITING PROTEIN 1* (*PGIP1*) gene is downregulated in *stop1* mutants which corroborated a previous report (Sawaki et al., 2009) showing that expression of *PGIP1* is downregulated in *stop1* and is involved in remodeling the cell-wall under low pH conditions by stabilizing the pectin network. This was later demonstrated in a report by Kobayashi et al. (2014) that showed that *pgip1* knock-out mutants have less cell wall stability and are more susceptible to root damage than the wild-type in response to low pH treatment. The previously mentioned reports indicate that cell wall stabilization, and specifically the modification of the pectin network, is involved in the tolerance to H^+^ and Al^3+^rhizotoxicities. Therefore, the study of cell-wall dynamics in response to H^+^ and Al^3+^ toxicities could provide valuable insights into the tolerance mechanisms of plants to conditions prevalent in acidic soils. Our analysis provides interesting candidate genes to continue the characterization of the role of cell-wall modifying enzymes and cell-wall carbohydrate dynamics in the root tolerance to H^+^ and Al^3+^ rhizotoxicities.

### RNA-Seq Provided Insights Into the Regulation Mechanism of STOP1

We guided our transcriptomic dissection using the turnover of the major transcriptional regulator of acid soil stress responses in Arabidopsis: STOP1. Our results indicate that low Pi, low pH, Fe excess and Al-exposure coordinately trigger STOP1-acummulation (**Figure 1**). Since we did not find *STOP1* to be differentially expressed in any of the treatments used in this study, our evidence supports the notion that STOP1 is regulated mainly at the posttranslational level. Previous research suggested that STOP1 is post-transcriptionally upregulated (Iuchi et al., 2008; Balzergue et al., 2017; Mora-Macías et al., 2017; Godon et al., 2019; Zhang et al., 2019) in response to stress conditions. Our data indicate that acidic stress signaling converges at two levels *via* STOP1 signaling: post-translationally through the regulation of STOP1 turnover and transcriptionally, *via* the activation of STOP1-dependent gene expression. A recent report corroborated post-translational regulation of STOP1 *via* the ubiquitin-proteasome pathway through interaction with F-box protein RAE1, establishing a feedback regulation loop of STOP1 turnover. A recent genetic dissection of responses to low Pi proposed that ALS3 functions upstream of STOP1 (Godon et al., 2019) because STOP1 over accumulates in *als3.* However, our results indicate that *ALS3* is regulated by STOP1. *ALS3* is within the group of STOP1-targets and its expression is upregulated in response to +Al-treatment in the wild-type but not in *stop1* (**Figure 5C**). In fact, it was previously reported that the expression of *ALS3* is controlled by STOP1 (Sawaki et al., 2009). Therefore, evidence indicates that RAE1 and ALS3 modulate the levels of STOP1 at the posttranslational level. Because STOP1 controls the expression of *RAE1* and *ALS3* at the transcriptional level, this suggests that at least two negative regulation feedback loops control STOP1 turnover in response to abiotic stress factors. It must be pointed out that, even though these two negative regulation feedback loops that control STOP1 turnover and are under STOP1 control have been identified, the initial activator of the STOP1-acummulation spike in response to stress conditions remains unknown.

Pleiotropy can be defined as the effect of one gene on multiple phenotypes. Multiple lines of evidence indicate that STOP1 has a an important role in response to multiple stresses including low pH, Al-toxicity, low Pi, hypoxia, salt-stress and drought tolerance (Iuchi et al., 2007; Balzergue et al., 2017; Mora-Macías et al., 2017; Enomoto et al., 2019; Sadhukhan et al., 2019). Therefore, mutations in STOP1 have a pleiotropic effect on the developmental and molecular responses to different abiotic stresses. The STOP1 pleiotropy can be explained in part because its target genes have important roles in response to multiple stresses (Magalhaes et al., 2018). This is the case of *ALMT1*¸ a STOP1-target that is essential for malate excretion, that plays a role in Al-exclusion (Hoekenga et al., 2006) and modification of root growth in response to low Pi conditions (Balzergue et al., 2017; Mora-Macías et al., 2017) and of *ALS3* that plays roles in Al-tolerance (Larsen et al., 2005) and modification of root growth in response to low Pi conditions (Dong et al., 2017). It has also been reported that STOP1 activates the expression of *GDH1*/*2* in response to hypoxia (Enomoto et al., 2019) and low pH conditions (Sawaki et al., 2009), which activates the GABA-shunt that regulates cellular H+ levels and prevents acidosis of the cytosol (Bouché and Fromm, 2004) in acidic and hypoxic environments. Given the pleiotropic role of STOP1, two main questions arise with respect to its activation mechanism: Does it respond to a single common signal, like a metabolite, ROS, or Ca^2+^ fluxes, or are there specific STOP1-regulation mechanisms for each type of stress? Given that STOP1-regulation appears to be mainly at the post-transcriptional level, it is most likely that STOP1 activity as a transcriptional regulator is modulated by interacting proteins under each type of stress. Nevertheless, further research regarding STOP1-regulation is required to answer these questions.

Using a dataset from a recent report on the global characterization of protein-cis-interactions, named the cistrome of Arabidopsis (O’Malley et al., 2016), we identified the set of STOP1-targets whose expression depends on STOP1-accumulation (**Figure 5**). We detected 76 genes whose expression is dependent on the level of STOP1 accumulation, including *RAE1* and *ALMT1.* However, we also found a large portion of the STOP1 identified targets by O’Malley et al. (2016) whose expression is not altered in *stop1*. Two possible scenarios could explain the latter: 1) STOP1 requires other transcription factors to be able to bind to the cognate binding site or to interact with the basal transcriptional machinery to activate transcription and 2) STOP1 is subjected to multiple posttranslational modifications that alter its affinity for different promoter sequences, like protein phosphorylation or sumoylation. Since STOP1 has orthologs in other species (Ohyama et al., 2013), the list of STOP1 target genes might provide new candidate genes to increase tolerance to acid soils. Further physiological and genetic engineering experiments are an exciting perspective that derives from the presented dataset.

## Data Availability Statement

RNA-Sequencing data reported in this article has been deposited in the Gene Expression Omnibus under the accession no. GSE148457 (https://www.ncbi.nlm.nih.gov/geo/query/acc.cgi?acc=GSE148457).

## Author Contributions

JOO-R and LH-E designed research. JOO-R and AO-A performed experiments. LH-E contributed reagents and analytic tools. JOO-R and LH-E analyzed data and JOO-R and LH-E wrote the paper.

## Funding

This work was supported in part by grants from the Basic Science program from CONACyT (Grant 00126261), the Governor University Research Initiative program (05-2018) from the State of Texas and by a Senior Scholar grant from Howard Hughes Medical Institute (grant 55005946) to LH-E.

## Conflict of Interest

The authors declare that the research was conducted in the absence of any commercial or financial relationships that could be construed as a potential conflict of interest.
