# Supplementary material for: Dissection of Root Transcriptional Responses to Low pH, Aluminum Toxicity and Iron Excess Under Pi-Limiting Conditions in Arabidopsis Wild-Type and stop1 Seedlings
Source: Front Plant Sci. 2020 Sep 29;11:01200. doi: 10.3389/fpls.2020.01200 (PMC7550639; doi:10.3389/fpls.2020.01200)
Supplement: Supplementary file 1 [file DataSheet_1.pdf]

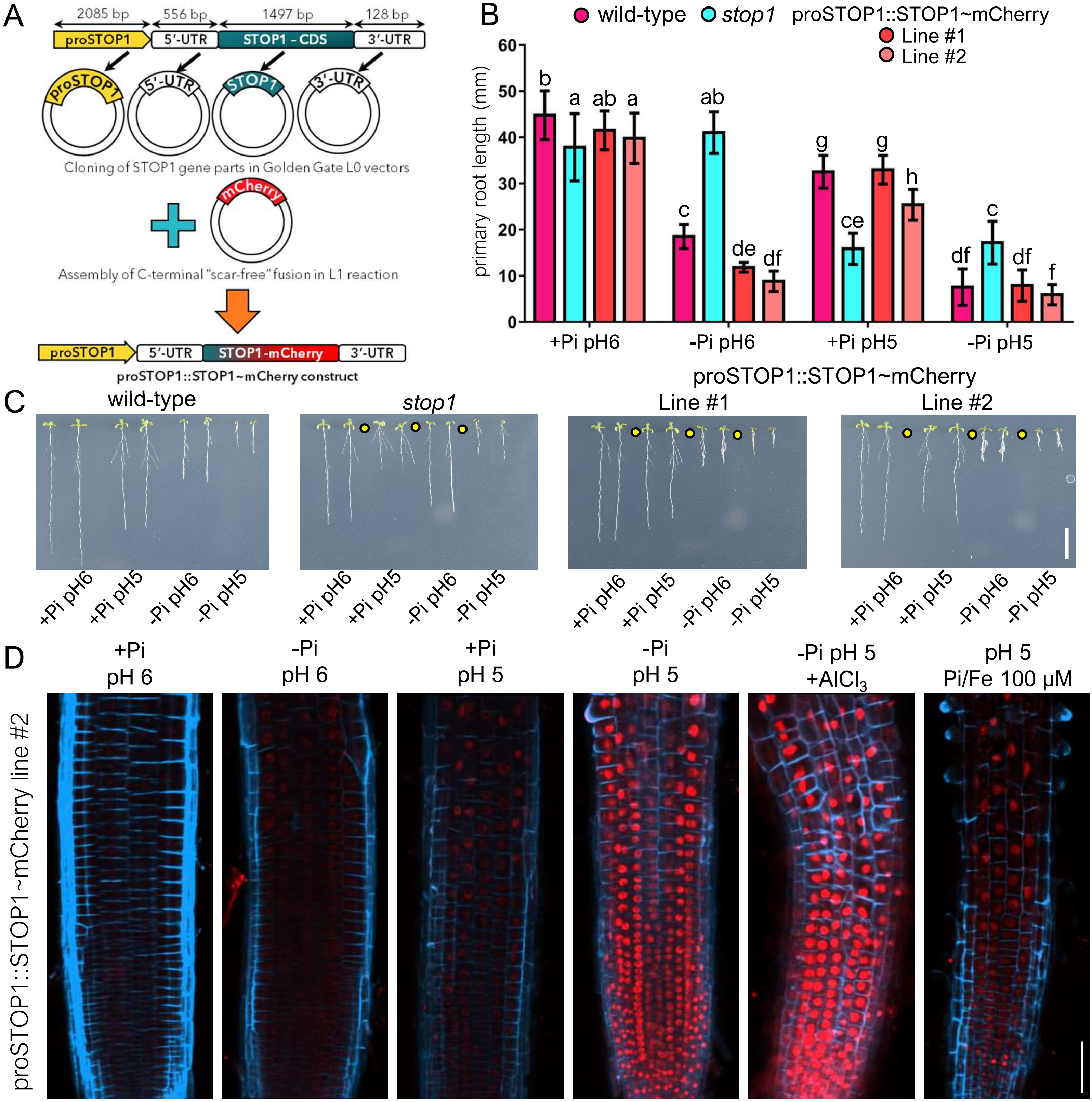

Supplementary Figure 1

The proSTOP1::STOP1~mCherry construct complements the stop1 mutant phenotype under low pH and low Pi conditions and is expressed in the nucleus of root cells in response to the conditions present in acidic soils.

A Depiction of the cloning strategy that was undertaken to generate the "scar-free" proSTOP1::STOP1~mCherry construct. See Materials and Methods for further details.

B Primary root length of wild-type, *stop1 and stop1* seedlings transformed with the proSTOP1::STOP1~mCherry construct (line#1 and line #2). Statistical groups were determined using a Tukey HSD test (P value <0.05) and are indicated with letters. Seedlings were grown 10 dag under the indicated conditions. Two biological replicates were performed with a total n=20 roots measured per genotype and condition tested.

C Phenotypes of wild-type, *stop1 and stop1* seedlings transformed with the proSTOP1::STOP1~mCherry construct. Seedlings were grown 10 dag under the indicated conditions. Scale bar equals 10 mm.

D Transgenic *stop1* seedlings expressing proSTOP1::STOP1~mCherry (line #2) were grown 5 days-after-germination (dag) and then transferred to the indicated medium conditions during 16h, at this time STOP1-mCherry signal (Red Channel) was observed using confocal microscopy, the cell-wall was stained using a modified DAPI-staining (see Materials and Methods). Scale bar equals 50 μM.

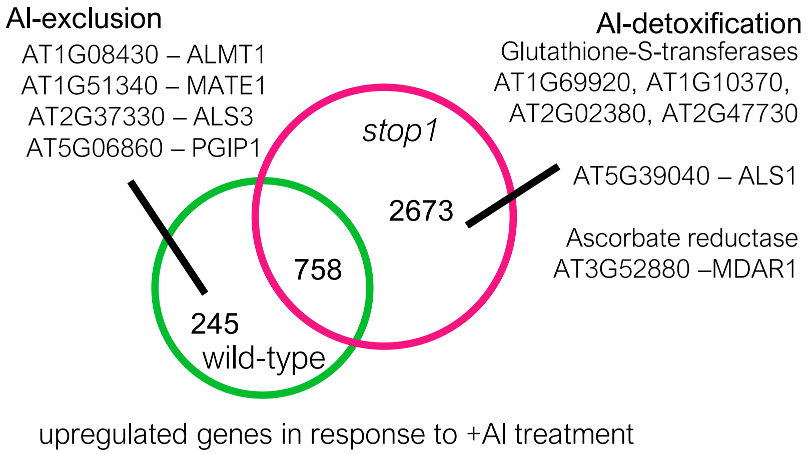

Supplementary Figure 2

Hypersensitive transcriptional response of Al-detoxification-related genes in the roots *stop1* seedlings in response to Al-treatment.

Venn diagram analysis of the genes that are upregulated in response to Al-treatment in the roots of wild-type and *stop1* seedlings in response to +Al-treatment. Examples of remarkable genes belonging to Al-exclusion and Al-detoxification processes (as defined by Kochian et al. 2015) are presented for the resulting specific subsets for wild-type and *stop1* seedlings, respectively.

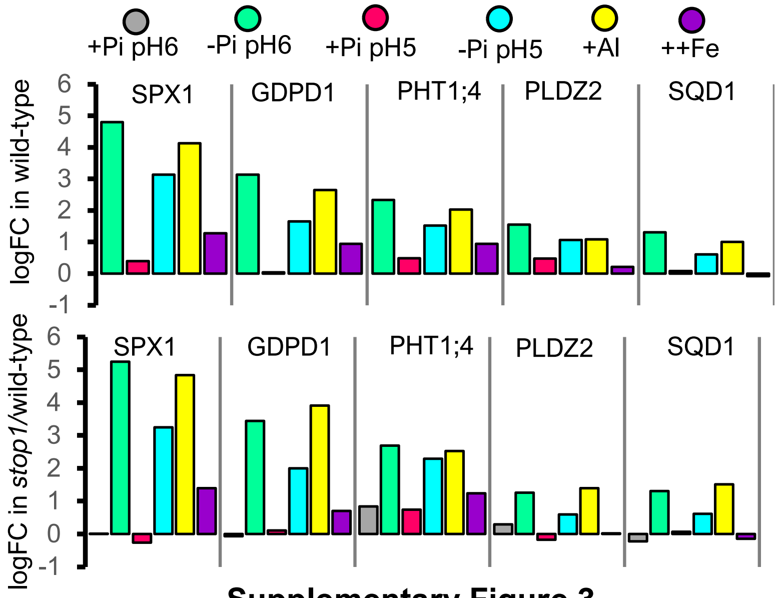

Supplementary Figure 3

Fe excess treatment does not induce the expression of low-Pi-inducible to the same extent that low Pi conditions at pH 5 and pH6

Changes in the expression in log2 of fold change (logFC) of PHR1-gene-targets in response to the indicated treatments with respect to the expression in control conditions (+Pi_pH6 treatment in Col-0).

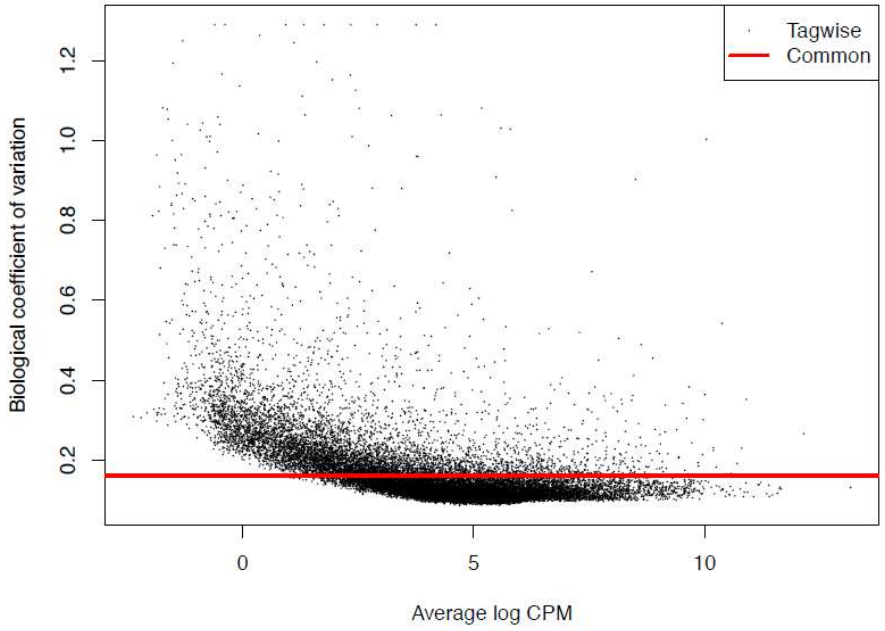

Supplementary Figure 4

Biological coefficient of variation of the 20,450 genes expressed in this RNA-seq study.

Biological coefficient of variation (BCV) of this study is highlighted in red. The BCV for each gene (tagwise) is represented, genes are ordered according to their expression level (in average log counts per million (cpm)).
